# Supplementary material for: Association between oral microbiome and five types of respiratory infections: a two-sample Mendelian randomization study in east Asian population
Source: Front Microbiol. 2024 Apr 10;15:1392473. doi: 10.3389/fmicb.2024.1392473 (PMC11039966; doi:10.3389/fmicb.2024.1392473)
Supplement: Supplementary file 1 [file Data_Sheet_1.zip › Supplementary Materials/MR plots of saliva/Bronchitis/s__Prevotella_melaninogenica_mgs_1984/leave_one_out.pdf]

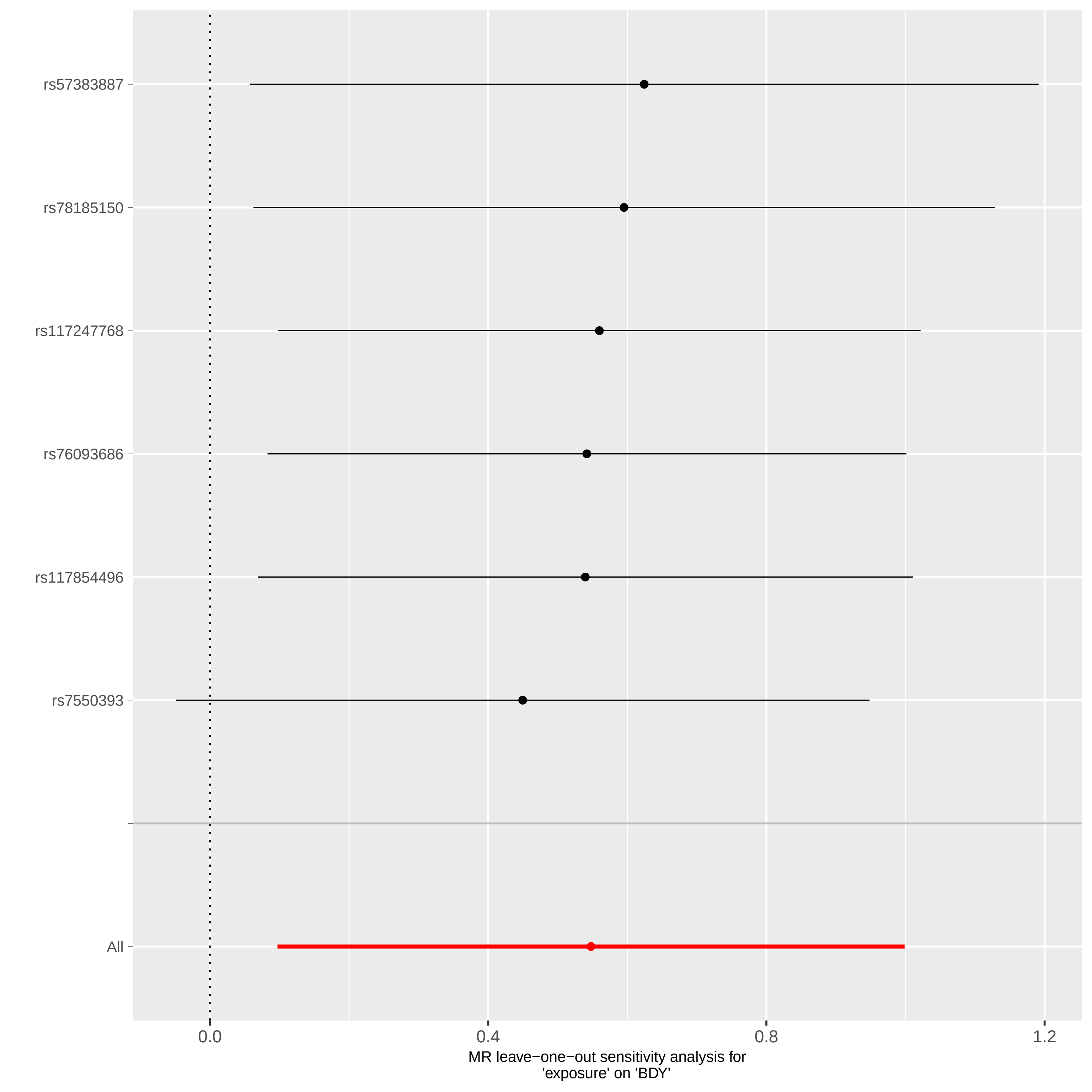
rs57383887
rs78185150
rs117247768
rs76093686
rs117854496
rs7550393
All
0.0
0.4
0.8
1.2
MR leave−one−out sensitivity analysis for
'exposure' on 'BDY'
